# Global Research Trends in Sports Nutrition and Football over the Last 20 Years (2004–2024)

**DOI:** 10.3390/sports13100365

**Published:** 2025-10-16

**Authors:** David Michel de Oliveira, Ana Karolina Assis Carvalho Silva, Anderson Geremias Macedo, Mayara Bocchi Fernandes, Eduardo Vignoto Fernandes

**Affiliations:** 1 Postgraduate Program in Health Applied Sciences, Unique Bioscience and Health Program, Immunometabolism, Nutrition, and Exercise Laboratory, Federal University of Jataí (UFJ), Jataí 75801-615, Goiás, Brazil; ana.karolina@discente.ufj.edu.br (A.K.A.C.S.); mayara.fernandes@ufj.edu.br (M.B.F.); eduardovignoto@ufj.edu.br (E.V.F.); 2 Institute of Motricity Sciences and Graduate Program in Rehabilitation Sciences, Federal University of Alfenas (UNIFAL), Alfenas 37130-001, Minas Gerais, Brazil; andersongmacedo@yahoo.com.br

**Keywords:** athletic performance, Nutrition and Sports Sciences, dietary intake, football athletes, Sports Medicine, Scientometrics, sports nutrition

## Abstract

Background: We aimed to map the scientific production on sports nutrition applied to soccer. Methods: A scientometric analysis was performed using articles published between 2004 and 2024, retrieved from Web of Science, PubMed, and Scopus. The search yielded 2636 documents, and 526 original articles were included after removing reviews, meta-analyses, duplicates, and studies outside the scope. Data were analyzed using Bibliometrix version 5.0.1; Massimo Aria & Corrado Cuccurullo; Naples; Italy. and VOSviewer version 1.6.20; Centre for Science and Technology Studies (CWTS), Leiden University; Leiden; The Netherlands software. Results: There was a 1.450% increase in publications over the period, with a peak in 2024. *Nutrients* was the leading publication source, while Morton J. and Maughan R. were the most productive authors. Liverpool John Moores University stood out as a collaboration hub. The United Kingdom 371 took the lead in both publication volume and citations. Early research trends focused on hydration and dietary optimization, whereas recent studies emphasized low energy availability, polyphenols, anthropometry, and recovery strategies. The conceptual structure focused on terms such as sports, nutrition, energy intake, food intake, performance, soccer, and training load. Peripheral terms included fluid balance and sweat rate. The co-occurrence analysis revealed underexplored topics such as oxidative stress, lipid peroxidation, beta-alanine supplementation, and antioxidant markers. Conclusions: Advancing these research areas is essential to consolidating nutritional strategies with direct effects on performance and health in soccer players.

## 1. Introduction

Sports nutrition, as a scientific field, represents an expanding area that integrates knowledge about nutrients, dietary practices, and their effects on physical performance [1]. Research in this field has generally adopted interdisciplinary approaches, involving nutrition, health sciences, biomedicine, sport, biochemistry, and exercise physiology, with the aim of understanding the physiological mechanisms related to optimizing athletic performance [2]. From a clinical perspective, sports nutrition plays a fundamental role in maintaining, recovering, and improving physical performance, contributing directly to maximizing results in training and competitions [3].

In this context, soccer stands out as one of the most widely practiced disciplines globally, with around 265 million players. According to data from the International Federation of Association Football (FIFA), between 2000 and 2006, there was a 9% increase in the number of male players and a 19% increase in female players [4].

Soccer is an intermittent sport that demands physical contact, rapid changes in direction, high-intensity actions, acceleration, deceleration, kicks, and sprints. These demands require agile motor responses, with combined use of strength, power, and aerobic endurance, considering that players travel long distances during a match [5]. This physiological profile results in high energy demand, with predominant activation of the aerobic and anaerobic pathways. Thus, adequate intake of macro and micronutrients, combined with specific nutritional strategies, is essential to sustain sports performance [6,7].

Although there is growing scientific interest in the areas of sports nutrition and soccer, the available studies tend to treat these themes in isolation, adopting different methods and scopes of analysis. Wu and Yin [8] analyzed scientific production related to the FIFA World Cup between 1974 and 2022, while Liu et al. [9] mapped global trends in youth soccer between 2012 and 2021. In the field of sports nutrition, Kiss et al. [2] examined publication trends between 2000 and 2018, and more recently, Abdullah et al. [10] provided an overview of the main directions in the field. Tao and Wu [11] also highlighted emerging topics at the interface between sport and nutrition. These isolated approaches, while contributing to the deepening of investigations in each area, still lack integrated works that examine the scientific outlook.

To date, there is no scientific mapping in the literature integrating the fields of sports nutrition and soccer, based on a survey of multiple indexing databases of high quality and global coverage, such as Web of Science, PubMed, and Scopus. In addition, these databases exhibit discrepancies between their coverage and information in the medical literature [12]. Therefore, a scientometric result that emerges from more than one database could offer a better understanding of the scientific landscape in this field.

Herein, by combining multiple literature databases, we aimed to carry out a scientometric mapping of sports nutrition applied to soccer and to identify knowledge gaps in the field. We sought to identify publication trends over the last 20 years, including journals, collaboration networks, authors, most cited documents, predominant keywords, and countries that have contributed most to the field. Through this approach, we mapped the current state of the art in the area and identified the main trends in its development. The central questions that will guide the investigation include the following: (1) What temporal trends and patterns of growth and/or decline can be identified in scientific production? (2) Which journals publish the most articles and are the most influential in scientific production in the area? (3) How are the collaboration networks structured between authors and institutions working in the field under investigation? (4) Which are the most cited studies and sources, and how do these citations contribute to the consolidation of knowledge in the area? (5) Which countries lead scientific production, and how is this leadership distributed globally? (6) What are the most recurrent conceptual structures and terms in scientific publications, and which themes stand out as a focus of interest or knowledge gaps in the field?

## 2. Materials and Methods

The current study uses scientometric analysis, an approach that quantifies the production of a specific scientific field, focusing on the analysis of original scientific articles. This methodology makes it possible to monitor the literature and identify trends [13].

### 2.1. Data Collection

Articles from the last 20 years, with publications from January 2004 to December 2024, were selected, as this time frame allows for mapping the most recent evolution of scientific production, identifying contemporary trends, and understanding emerging research lines at the interface between nutrition and soccer. The following strings combined with Boolean operators were used as the search strategy: “(“nutritional status” OR “nutrition assessment” OR “dietary intake” OR “sports nutrition” OR “macronutrient intake” OR “micronutrient intake” OR “energy availability” OR “hydration” OR “supplementation”) AND (“soccer” OR “football” OR “soccer players” OR “football players” OR “elite soccer” OR “professional football” OR “youth soccer” OR “Women’s football” OR “women’s soccer”)”.

To retrieve the papers, we used the following databases: Web of Science (WoS) (https://www.webofscience.com URL (accessed on 3 March 2025), PubMed (https://pubmed.ncbi.nlm.nih.gov URL (accessed on 4 March 2025) and Scopus (https://www.scopus.com/home.uri. URL (accessed on 4 March 2025). The files from the three databases were merged using the Bibliometrix 4.1.3 package [14] implemented in R 4.4.2 (R Core Team, 2024). Reviews and meta-analyses were removed from the analysis. Duplicate articles were also removed using Bibliometrix 4.1.3 [14].

**Figure 1:**
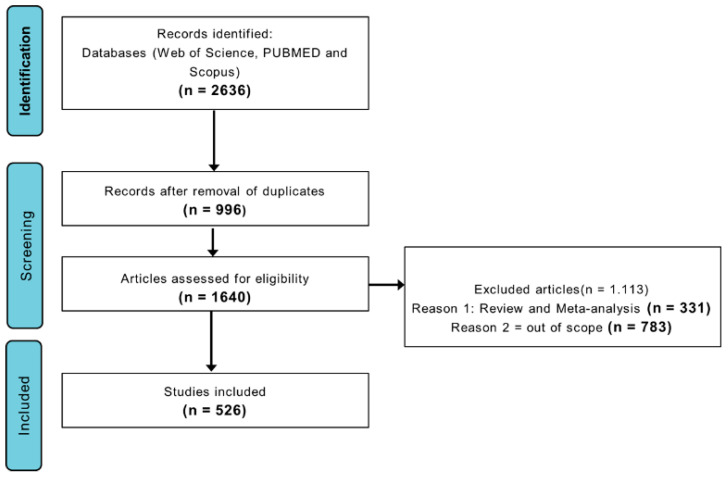
Flowchart adapted from the PRISMA protocol [15] identifying the stages of screening and eligibility of scientific articles on sports nutrition applied to soccer.

Studies on field soccer played by men and women of all age groups were included. We used exclusion criteria of works related to Australian soccer, American soccer, rugby, Gaelic soccer, futsal, adapted soccer, soccer referees, and other sports that did not meet the scope of the investigation. After filtering and eligibility, the final database consisted of 526 publications (Figure 1).

### 2.2. Scientometric Analysis

Descriptive analyses and scientific mapping were conducted using the Bibliometrix 4.3.2 package [14] implemented in R 4.4.2 (R Core Team, 2024).

We generated a graph of annual scientific production to visualize the temporal evolution of publications in the field. To identify the most active researchers and their contributions over the years, we analyzed the authors’ output over time.

To investigate trends in publications in the journals, we carried out a three-field plot analysis through the Sankey diagram, configured with 10 items in the fields of keywords, scientific journals, and authors. To examine the distribution of articles in the journals and identify the journals that contribute most to the area, Bradford’s Law of Dispersion analysis [16] was applied. For the investigation of the total number of citations and the average number of citations per year, we analyzed the most cited documents locally, also using Bibliometrix.

To visualize research partnerships in the field, we analyzed the collaboration network between authors and institutions. Authors with at least two published documents were included. Trends and patterns of international collaboration and co-authorship between authors were established through the application of the analyses of Local Scientific Production of Countries, Production of Countries Over Time, and Most Cited Countries, using Bibliometrix.

To assess thematic trends in the field over time, we used the author’s keywords associated with the year of publication. To visualize the structure of knowledge in the field, we generated the conceptual structure map using the author’s keywords in order to better understand the theme concepts and their connections.

To gain a deeper understanding of the field and to identify thematic clusters and how they connect with each other, we performed a co-occurrence network with authors’ keywords analysis, also using VosViewer, version 1.0.0 [17]. As inclusion criteria to perform the analysis, we used terms that appear together in the publications.

## 3. Results and Discussion

In the period of the survey of publications on sports nutrition applied to soccer (2004–2024), we found a total of 526 documents, distributed in 197 scientific journals, with an annual growth rate of 14.6%. The analysis identified a total of 2478 authors in the field, of which 15 are authors of single-authored articles. On average, there are 6.04% co-authors per document, and 23.9% of publications resulted from international collaborations. Each scientific article has an average of 15.4 citations. Table S1 presents the complete list of articles that make up the sample analyzed in this study.

### 3.1. Temporal Trends in Publication and Authorship

**Figure 2:**
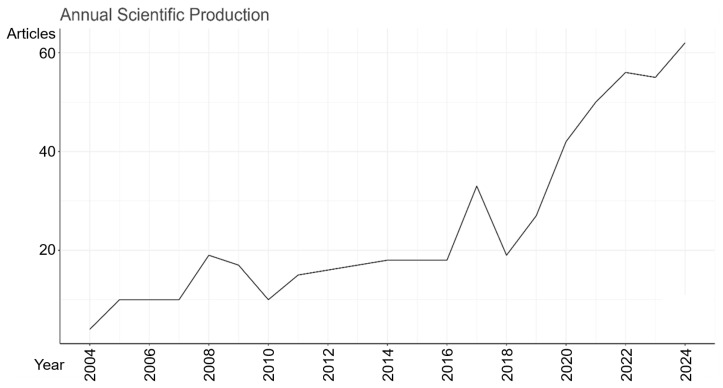
Number of scientific articles published between January 2004 and December 2024 on sports nutrition applied to soccer.

There was a decline in production between 2008 and 2010, variations between 2012 and 2018, followed by continuous growth from 2020 onwards. In 2004, which corresponds to the first year of our time frame, four articles were published, while in 2024, there were 62, an increase of 1.450%, confirming the increase in research on the subject over the last two decades (Figure 2). This trend converges with the findings of Liu et al. [9], who also reported an increase in scientific production related to youth soccer in the period from 2012 to 2021.

The World Cup is one of the main sporting events in this field, and can stimulate academic interest, especially in research on sports performance [8]. In this sense, we hypothesized that the years before or after the World Cup competitions could influence and increase the number of publications. However, the data analyzed do not indicate an increase in scientific production on sports nutrition applied to soccer. In the World Cup years (2006, 2010, 2014, and 2018), there was stability or even a drop in the number of publications in the field of sports nutrition applied to soccer, suggesting that scientific activity was not boosted by these events. From 2020 onwards, however, there has been an increase in annual production following the pandemic scenario [18].

The scientific output of the main authors in the field presents an asymmetrical distribution over time, with some researchers concentrating on their publications in more recent periods, while others stand out in the early stages of the time frame analyzed. Figure 2 shows the scientific output of the main authors over the time period investigated. The size of the bubbles indicates the number of articles published per year, and the intensity of the color reflects the impact of the publications (average number of citations per year).

The authors Morton J (n = 12) and Maughan R (n = 10) have the highest number of publications, but the production of the author Morton J intensified from 2016 until 2024. Authors such as Russell M (n = 09) and Rollo I (n = 07) also show increasing activity from 2018 onwards. The author Dvorak J (n = 08) is highlighted for the high average impact of citations per year of his publications; in 2008, he reached 326 citations and an average of 18.1 citations/year with articles on the line of research on the effect of fasting in Ramadan on the nutritional behavior, health, and performance of players [19,20,21,22]. In 2010, Dvorak J obtained 137 citations and an average of 8.56 citations/year with an article on heat stress and hydration in soccer [23].

**Figure 3:**
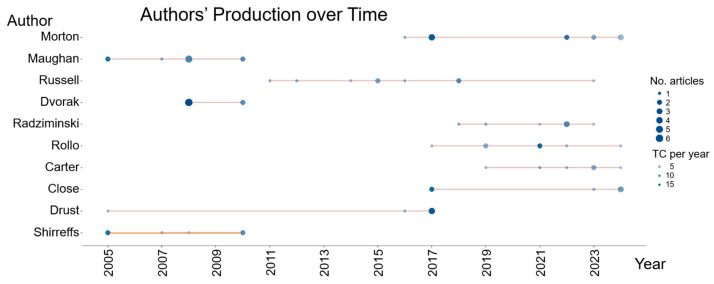
Scientific production of the main authors over time on sports nutrition applied to soccer. Caption: TC: Total Citations.

Dvorak J was named as the most productive author in a bibliometric study on scientific production related to the FIFA World Cup between 1974 and 2022 [8], reinforcing his relevance on the international soccer research scene. On the other hand, among the authors who have contributed the most to the field, we identified Shirreffs S, with relevant output between 2005 and 2010 (12.3 citations/year), but who did not continue to publish in the field in the following years (Figure 3).

### 3.2. Trends in Scientific Journals

**Figure 4:**
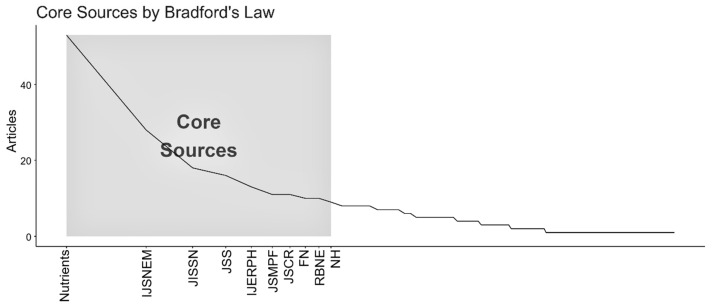
Bradford’s Dispersion Law [16]. The figure shows the distribution of journals in productivity zones. Zone 1 (core sources) brings together the journals with the highest number of publications on sports nutrition and soccer in the period analyzed, while the subsequent zones concentrate on journals with a lower frequency of publications on the subject. Caption: IJSNEM = *International Journal of Sport Nutrition and Exercise Metabolism*; JISSN = *Journal of the International Society of Sports Nutrition*; JSS = *Journal of Sports Sciences*; IJERPH = *International Journal of Environmental Research and Public Health*; JSMPF = *Journal of Sports Medicine and Physical Fitness*; JSCR = *Journal of Strength and Conditioning Research*; FN = *Frontiers in Nutrition*; RBNE = *Revista Brasileira de Nutrição Esportiva*; NH = *Nutrición Hospitalaria*.

Figure 4 shows Bradford’s Dispersion Law [16], demonstrating the organization of journals into productivity zones. The journals highlighted in “gray” make up the core source with the highest productivity in sports nutrition applied to soccer over the last 20 years.

There is strong centralization in a few journals, with the journal *Nutrients* taking the lead in the number of publications. Although Nutrients [24] is an open-access journal, with an impact factor of 4.8, the high concentration of studies in this journal may reflect not only its broad thematic coverage, including a section dedicated to sports nutrition, but also the authors’ strategy of prioritizing journals with greater reach and ease of dissemination.

The distribution of scientific production in scientific journals, organized in order of the journals that have published the most in the field, containing the absolute and relative values (%), as well as the impact factor of each one, is shown in Table 1.

The results of the current study converge with the findings of Abdullah et al. [10], who mapped scientific production on sport and nutrition using the WOS and Scopus databases. In both analyses, the journals *Nutrients*, *International Journal of Sport Nutrition and Exercise Metabolism*, and *Journal of the International Society of Sports Nutrition* occupy, respectively, the top three positions in terms of volume of publications, reinforcing their centrality in the dissemination of sports nutrition research.

The Sankey Diagram (Figure 4) shows the flow of information between three interconnected fields: descriptors, journals, and authors [26]. The terms “soccer”, “football”, and “nutrition” are the main descriptors in the field. The journals *Nutrients* [24] and *Journal of Sports Sciences* [27] were the main vehicles for scientific dissemination during the period investigated, while the *Brazilian Journal of Sports Nutrition* [28] also stands out, highlighting production in South America, especially in Brazil. Although its metrics are not high, it is indexed in the Web of Science [29], has no submission fee, and accepts papers in several languages, which contributes to the dissemination of knowledge in the country.

**Figure 5:**
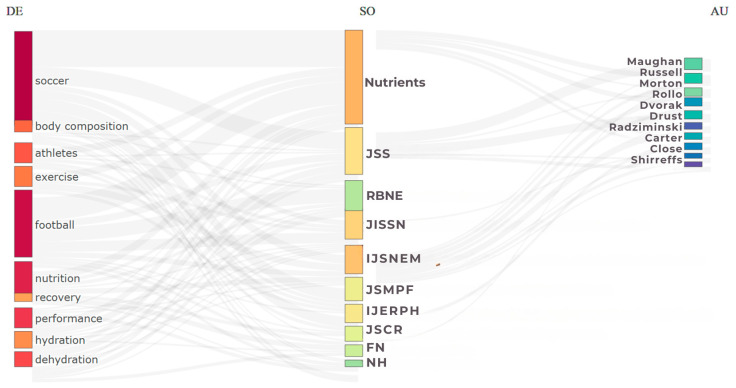
Sankey diagram. Graph of three fields: keywords (DE), scientific sources (SO), and authors (AU). Caption: JSS = *Journal of Sports Sciences*; RBNE = *Revista Brasileira de Nutrição Esportiva*; JISSN = *Journal of the International Society of Sports Nutrition*; IJSNEM = *International Journal of Sport Nutrition and Exercise Metabolism*; JSMPF = *Journal of Sports Medicine and Physical Fitness*; IJERPH = *International Journal of Environmental Research and Public Health*; JSCR = *Journal of Strength and Conditioning Research*; FN = *Frontiers in Nutrition*; NH = *Nutrición Hospitalaria*.

The authors Maughan R, Russell M, and Morton JS have strong links with the themes and journals identified in this field of research (Figure 5).

Table 2 presents the most cited documents, with a total of 1113 citations. Edwards et al. [30] stand out as the most cited, with 14.5% of the citations. The first 3 authors account for 38.3% of the citations. The study by Edwards et al. [30] analyzed the impact of moderate dehydration on the physical and mental performance of soccer players after 45 min of play in an outdoor environment. The results showed that even a slight loss of water significantly compromised physical performance and mental concentration. This work has become highly cited for being one of the first to demonstrate, in real game conditions, the relevance of hydration for maintaining integrated performance (physical and cognitive) in soccer. The contribution of this study highlighted relevant gaps in the literature, suggesting the need for individualized hydration strategies and protocols that consider not only physical performance but also the mental aspects of athletes, especially in situations of heat stress.

### 3.3. Partnerships in the Scientific Field: Collaborative Networks Between Authors and Institutions

**Figure 6:**
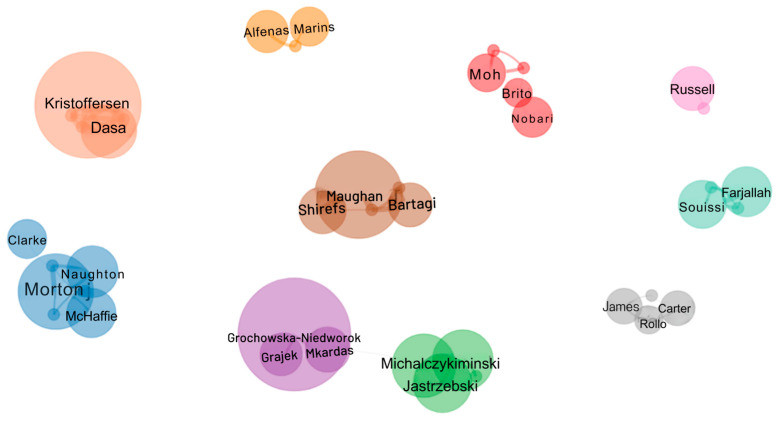
Collaboration networks between authors on sports nutrition applied to soccer.

The collaboration networks between authors were made up of 10 distinct clusters, totaling 33 authors distributed in groups of varying sizes and densities (Figure 6). It can be seen that there is no collaboration between the groups. There is a division in the scientific collaboration, with a predominance of interactions concentrated in small nuclei, which correspond to clusters x, y, and z. This characteristic is common in areas where the production of knowledge is structured around specific or thematic groups, defined as restricted collaborations directed by institutional links or thematic affinities [26].

The brown cluster of authors Maughan R, Morton J, Grochowska-Niedworok E, and Kristoffersen M has the largest collaboration network. In contrast, there are smaller and less connected groups, such as Russell M (pink cluster) or James L (gray cluster), which point to smaller interactions. This pattern, also observed by Liu et al. [9], indicates low integration between groups. Although Tao and Wu [11] identified specific collaboration groups using VOSviewer [17], the networks remain fragmented, which highlights the difficulty of articulation between different authors in the field of sports nutrition applied to soccer. This loosely articulated structure between groups can reduce the exchange of knowledge and limit the development of the field [40]. Among the possible causes of this fragmentation are the concentration of production in thematic clusters, influenced by funding limitations, and the applied nature of the field, which often directs research toward local or regional demands. As recommendations, we highlight the need to foster multicenter and international collaborations, encourage the formation of thematic consortia in sports nutrition, and expand the use of open data platforms to strengthen global integration. These initiatives can not only enhance knowledge exchange and the generation of more robust evidence but also support the development of practical guidelines and more consistent policies in the field of sports nutrition applied to soccer.

**Figure 7:**
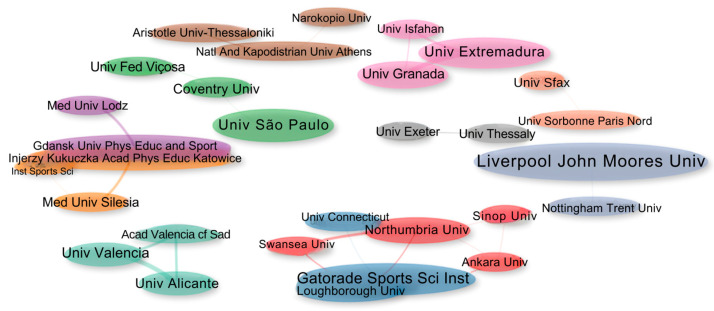
Collaboration networks between institutions. Each node represents a research institution. The size of the nodes is proportional to the number of publications and the thickness of the lines between the nodes to the strength of the collaboration between the institutions.

The collaboration networks between institutions are represented in Figure 7 and show eight clusters, made up of 30 institutions that published at least two articles related to Sports Nutrition applied to Football. The institutional collaboration networks showed a clearly structured geographical distribution in regional poles of scientific production.

Liverpool John Moores University stood out as the main international center of collaboration (gray cluster). In Latin America, the University of São Paulo (USP) led the regional connections (dark green cluster), establishing collaborations with the Federal University of Viçosa (Brazil) and Coventry University (UK). USP was also ranked 2nd as an institution researching soccer in the findings of Wu and Yin [8].

In Central Europe, dense, interconnected networks were observed (purple and orange clusters), led by Polish institutions such as the Gdansk University of Physical Education and Sport and the Jerzy Kukuczka Academy of Physical Education in Katowice. In the Iberian Peninsula, the light green cluster articulated between the University of Valencia and the University of Alicante stands out, exemplifying thematic networks associated with performance sport, as pointed out by Tao and Wu [11], who identified the formation of scientific clusters organized by areas of specialization in international collaborative networks in the area of sport and nutrition.

The work of the Gatorade Sports Science Institute (dark blue cluster), a reference organization in research applied to sports performance, reinforces the rapprochement between academic research and professional practice. These findings show consolidated regional collaborations and the advance of internationalization in scientific production in the area.

### 3.4. Scientific Output per Country

Among the articles analyzed on Sports Nutrition applied to Football, with 46.6% of total scientific production, the United Kingdom stood out as the country with the greatest scientific contribution, accounting for the majority of total production, followed by Brazil (42%) and Spain (38.2%). This pattern of geographic distribution of scientific production partly reflects the sporting prominence of these countries in soccer. The United Kingdom, historical cradle of the sport and home to one of the most competitive leagues in the world (Premier League), maintains scientific production aligned with sports nutrition in soccer [36,41]. Brazil, traditionally recognized as a soccer powerhouse, has significant scientific output in the field of sports science [42].

Spain, with its strong club structure and investment in science applied to high performance, also stands out as a scientific hub in the area [43]. Thus, there is a convergence between sporting excellence and scientific production, suggesting that the technical development of sport in these countries goes hand in hand with the generation of qualified scientific knowledge. On the other hand, there are no countries from South America (except Brazil), Africa, or Southeast Asia, indicating a geographical gap.

The percentages indicate the participation of each country based on the authors’ affiliation, as shown in Table 3. As several articles involve international co-authorship, the same study can be accounted for by more than one country, reflecting the collaborative nature of research in the area.

These results partially converge with the findings of Wu and Yin [8], who identified the United Kingdom in first place (over 400 articles), the United States in second place (386), and Brazil in third place (296). The higher values found by those authors can be explained by the fact that the authors used a bibliometric approach, which includes other scientific products as well as original articles. In addition, our findings were screened for specific sub-areas, a method not found in the aforementioned study.

**Figure 8:**
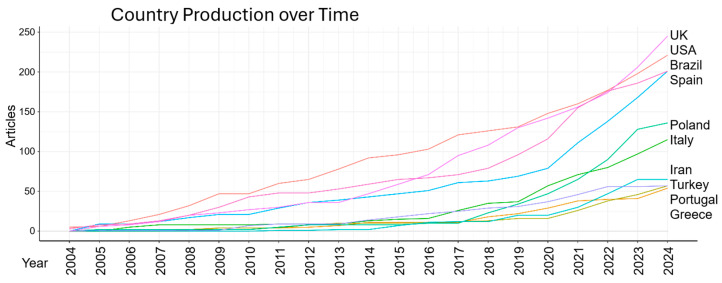
Country production over time.

In line with the previous results, the United Kingdom and Brazil lead the volume of publications, over the years, with a marked increase from 2015 onwards (Figure 8). The United States and Spain follow, with Poland showing an increase after 2017. Italy, Iran, Portugal, Turkey, and Greece also present increases in production over time, although less than the leaders.

Based on the total number of citations among the ten most cited countries (totaling 6250 citations), it can be seen that the United Kingdom accounts for 29.5% of this total. This leadership can be attributed to factors such as research promotion policies, consolidated academic infrastructure, scientific production aimed at practical application, and integration between universities and the sports sector [44]. In addition, there is a strong application of scientific results in real contexts, especially in high-performance sports, which increases the relevance and impact of the research developed [45].

**Figure 9:**
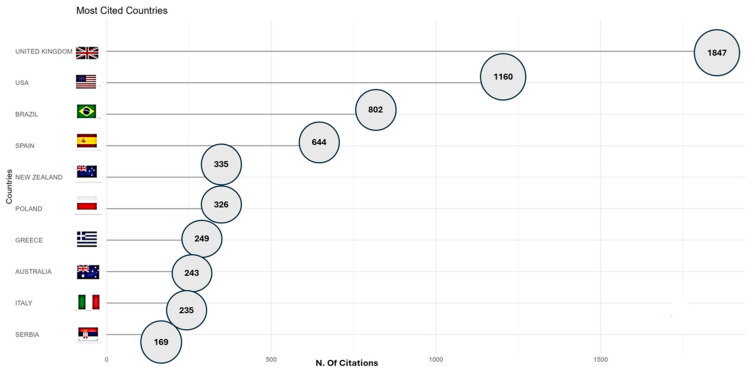
Most cited countries.

The United States follows with 18.6%, while Brazil and Spain contribute 12.8% and 10.3% of the citations, respectively. The other countries have less than 5.5% (Figure 9). Our results reinforce the role of the United Kingdom [8] as a consolidated center of scientific production and dissemination, as a result of the body of production in sports research and strong insertion in international networks. However, Liu et al. [9], when analyzing the literature on youth soccer using bibliometric analysis, identified the United States as the most cited country, followed by the United Kingdom. This inversion in the rankings can be explained by methodological differences, such as the time frame of the period analyzed, which can directly affect the volume and type of production in each country [9].

### 3.5. Structure and Trends of Themes in the Scientific Field

**Figure 10:**
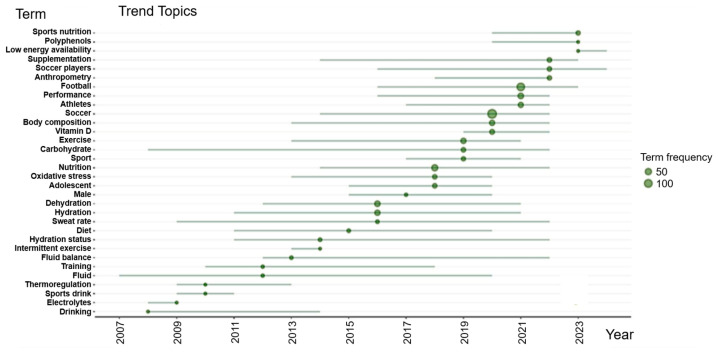
Trending topics in sports nutrition applied to soccer (2004–2020). Each circle corresponds to the frequency of the term over the years.

The temporal analysis of the topics shows changes in the frequency and focus of the themes researched in sports nutrition applied to soccer over the period analyzed. Figure 10 presents the distribution of topics mentioned in scientific publications and their temporal trends over the 20-year period.

In the initial trends (2006–2012), terms related to hydration predominated, such as “thermoregulation” [46], “sports drink” [47], “electrolytes” [48], and “fluid” [49]. This pattern indicates that, during this period, the focus of the field was mainly on the study of fluid loss and its replacement during sports practice.

From 2013 onwards, the terms “hydration status” [50], “diet” [51], “carbohydrate” [52], “exercise” [53], and “oxidative stress” [54] began to occur more frequently. This period marks a broadening of the focus on hydration to include nutritional aspects related to the metabolic demands of exercise, such as macronutrient intake, energy balance, and the impact of oxidative stress induced by physical exertion.

Between 2018 and 2020, the terms were consolidated: “soccer”, ‘football, athletes’, and ‘performance’, highlighting the direct interaction of the nutritional area with performance in soccer. As these terms are ubiquitous and central to the subject of sports nutrition in soccer, they are treated as specific elements of the field.

The terms: “body composition” [55], “vitamin D” [56], and “supplementation” [57] also appear more frequently, indicating growing interest in the influence of body composition and nutritional supplements.

Recent trends (2021–2024) have seen more emerging and strategic topics, such as “low energy availability” [58] and “polyphenols” [59]. Polyphenols have been investigated for their ability to mitigate exercise-induced oxidative stress by neutralizing free radicals, modulating inflammatory pathways, and supporting immune function. From a practical perspective, polyphenol-rich nutrients have been associated with improved recovery, reduced muscle damage, and enhanced performance in high-intensity efforts, which are common in soccer. These mechanisms and applications reinforce the relevance of this emerging topic and highlight the need for future studies to establish evidence-based strategies that connect nutritional interventions with performance outcomes in soccer players [59].

The topics of “soccer players” and “anthropometry” appear recurrently throughout the period analyzed, as they represent, respectively, the main object of study in the area and one of the most widely used tools for indirectly assessing nutritional status. In general, the topics identified point to a maturing of the field, focusing on nutritional individualization, recovery, and the prevention of energy deficiencies in players. The field of Sports Nutrition applied to Football has evolved from more basic topics, such as hydration, to more complex and specific areas, such as energy balance and nutritional supplementation, suggesting a growing alignment of research into personalized nutritional interventions, metabolic recovery, and the prevention of energy deficiencies.

The analysis of uniterms makes it possible to identify both the thematic structure and connections between the main terms used in the literature.

**Figure 11:**
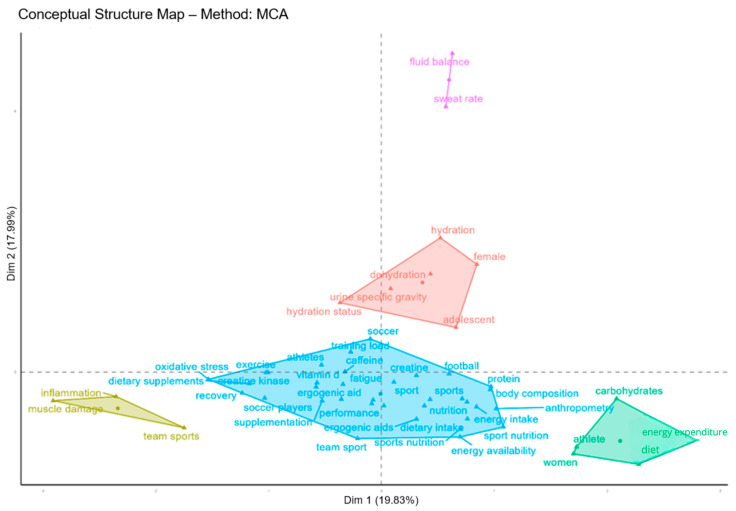
Conceptual Structure Map (CSM).

The conceptual structure map (Figure 11) and the co-occurrence network (Figure 11) are complementary analyses: while the former reveals the conceptual organization of the field, the latter shows the frequency and association between terms in publications [14,17].

The visual representation of the conceptual structure of the field groups related themes into 5 clusters, considering the articles analyzed (Figure 10). The X axis (Dim 1) explains 19.83% of the variance in the data, while the Y axis (Dim 2) explains 17.99%, totaling around 38% of the variance explained.

Terms that are close to each other form thematic groupings (clusters), indicating that they are often mentioned together. The blue clusters make up the central axis of research on “Nutrition and Performance in Football”, with terms such as: “sports nutrition”, “energy intake”, “dietary intake”, “performance”, “football”, and “training load”, indicating that research in this area focuses mainly on nutritional intake and physical performance during soccer practice.

The red clusters focus on hydration in specific populations, such as “hydration”, “dehydration”, “female”, “adolescent”, and “hydration status”, highlighting the growing attention given to studies on hydration in women and adolescents.

The green cluster points to research into body composition, energy requirements, and gender differences (“athlete”, “women”, “carbohydrates”, “diet”, “energy expenditure”, “anthropometry”), suggesting a focus on nutritional strategies according to the physiological profile and metabolic needs of different groups of athletes.

The yellow cluster includes a line of research pointing to “inflammation”, “team sports”, and “muscle injuries”, indicating a focus on injury prevention and recovery from muscle injuries using nutritional strategies. Finally, the purple cluster has a lower proportion in the peripheral axis, but with specificities indicating a conceptual field in the area of “fluid balance” and “sweat rate”. These terms indicate a conceptual field focused on hydration control and sweat rate and their influence on performance.

Our findings show that the conceptual field of sports nutrition applied to soccer is structured around its application to performance and has been expanding into specific sub-areas, with concepts aimed at studies with different athlete profiles and the development of individualized nutritional strategies for the athlete.

**Figure 12:**
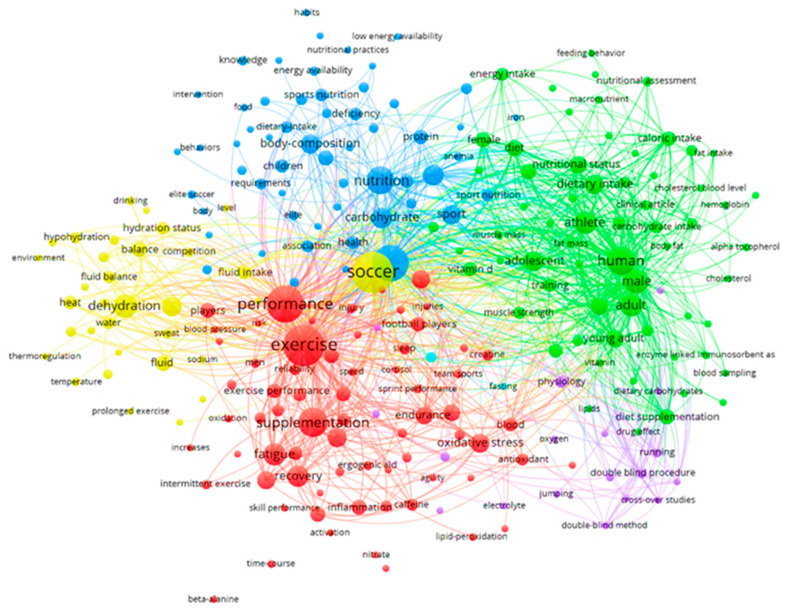
The co-occurrence network with the authors Keywords on Sports Nutrition applied to soccer. The size of the circle indicates its frequency, and the thickness and proximity of the lines show their association. The colors identify thematic groups, which can be seen as the main sub-areas of the field. Table S2 contains the frequency and distribution of keywords extracted from the metadata of the articles.

The author’s keyword co-occurrence network identified five clusters, including topics that frequently appear together in publications (Figure 12).

The red cluster contains the most frequent and central terms (“exercise”, “performance”, and “supplementation”), which demonstrate stability and relevance over time. The terms “exercise” and “performance” were also identified by Tao and Wu [11] as central terms in their analysis of the period from 2013 to 2023, reinforcing the thematic consistency, although different methodologies were used to reinforce the structuring axis of research in the area.

The green cluster groups words related to the diet and profile of practitioners, such as “athlete”, “male”, “adult”, and “young adult”, highlighting different populations. The blue cluster concentrates the terms “nutrition”, “carbohydrate”, and “body composition”, demonstrating the integration between nutritional intake and body composition parameters.

The purple cluster, located at the right end of the concept map and with the lowest density, is related to experimental methods and laboratory evaluations. The less frequently used terms: “beta-alanine”, “lipid peroxidation”, “alpha tocopherol”, “enzyme linked immunosorbent assay”, and “low energy availability”, are peripheral terms to the center of the network, which have fewer links and combinations with other keywords, and low occurrence in the articles, and that partially explored the theme of oxidative stress, while other topics, such as supplementation with “beta-alanine” and issues related to “low energy availability”, were also less commonly addressed.

“Low energy availability” is a condition in which energy intake is insufficient to meet exercise demands and maintain physiological functions. Further research into this condition is essential, as it contributes to the prevention of immunosuppression, hormonal changes, increased risk of injury, and loss of performance in athletes [60,61]. The topics found, although isolated, contribute to understanding the antioxidant response to exercise and the need for specific supplementation strategies in soccer players [56,59,62,63].

Supplementation with beta-alanine, a precursor of muscle carnosine that acts as an intramuscular buffer against hydrogen ion accumulation, has been associated with improved tolerance to high-intensity exercise, as observed in the study by Rosas et al. [64], reinforcing its importance for anaerobic performance, predominant in some technical soccer positions.

Continued research along these lines into low energy availability and beta-alanine supplementation could contribute to preventing fatigue and immunosuppression and optimizing anaerobic performance. These themes also lack greater representation in specific nutritional guidelines, indicating the need for research that articulates evidence with practical recommendations for different athlete profiles.

### 3.6. Study Limitations

Some limitations should be considered when interpreting the results. The selection of databases, the exclusion of gray literature and non-indexed journals, may not have covered all relevant publications on the subject. In addition, the time frame adopted offers only a specific view of the period analyzed. Future research may expand coverage to other databases and adopt longer intervals, providing a more comprehensive understanding of scientific production.

## 4. Conclusions

Although progress has been made in the field of sports nutrition, some lines of research remain largely unexplored in the context of soccer. The analysis of the co-occurrence of keywords shows that the topics of lipid peroxidation, alpha-tocopherol, and the use of the enzyme-linked immunosorbent assay method, all related to oxidative stress, have been little investigated. These investigations help in the process of muscle recovery and performance. Furthermore, topics such as low energy availability and beta-alanine supplementation, which are essential for maintaining nutritional status and improving energy resynthesis, still require further study.

Continued research along these lines could strengthen evidence-based nutritional strategies, with a direct impact on footballers’ performance and health. It is, therefore, recommended that future research explores these topics, with a view to improving the performance and health of soccer players.

## Figures and Tables

**Table 1:** Percentage distribution of publications per journal according to Bradford’s Law and the impact factor of journals according to JCR (2023).

Journal	No. of Articles	% of Total	IF (JCR 2023)
1st *Nutrients*	53	29.6%	4.8
2nd *International Journal of Sport Nutrition and Exercise Metabolism*	28	15.6%	4.6
3rd *Journal of the International Society of Sports Nutrition*	18	10.1%	3.0
4th *Journal of Sports Sciences*	16	8.9%	2.8
5th *International Journal of Environmental Research and Public Health	13	7.3%	1.4
6th *Journal of Sports Medicine and Physical Fitness*	11	6.1%	1.2
7th *Journal of Strength and Conditioning Research*	11	6.1%	2.0
8th *Frontiers in Nutrition*	10	5.6%	6.0
9th *Revista Brasileira de Nutrição Esportiva*	10	5.6%	0.2
10th *Nutrición Hospitalaria*	9	5.0%	1.5

Source: Journal Citation Reports [25]. Accessed: April 2025; Removed: Impact factor removed in 2023*. Clarivate has discontinued indexing of the *International Journal of Environmental Research and Public Health* in Web of Science.

**Table 2:** Most cited documents (2004–2024) on Sports Nutrition Applied to Soccer.

Ranking/Author/Year	Source	TC	TC per Year
1st Edwards A, 2007 [30]	*British Journal of Sports Medicine*	161	8.47
2nd Shirreffs S, 2005 [31]	*International Journal of Sports Medicine*	147	7.00
3rd Ali A, 2007 [32]	*Medicine and Science in Sports and Exercise*	119	6.26
4th Tscholl P, 2008 [33]	*British Journal of Sports Medicine*	114	6.33
5th Maughan R, 2005 [34]	*Journal of Sports Sciences*	112	5.33
6th Hoffman J, 2008 [35]	*Nutrition Research*	105	5.83
7th Anderson L, 2017 [36]	*International Journal of Sport Nutrition and Exercise Metabolism*	101	11.22
8th Micheli M, 2014 [37]	*International Journal of Sports Physiology and Performance*	87	7.25
9th Devlin B, 2017 [38]	*International Journal of Sport Nutrition and Exercise Metabolism*	84	9.33
10th Bandelow S, 2010 [39]	*Scandinavian Journal of Medicine & Science in Sports*	83	5.19

Legend: TC: Total Citations.

**Table 3:** Scientific Production of Countries on Nutrition Applied to Football.

Ranking	Country	Number of Articles	Percentage of Total Production
1	United Kingdom	245	46.6%
2	Brazil	221	42.0%
3	Spain	201	38.2%
4	Usa	201	38.2%
5	Poland	136	25.9%
6	Italy	115	21.9%
7	Portugal	65	12.4%
8	Iran	57	10.8%
9	Turkey	57	10.8%
10	Greece	54	10.3%

## Data Availability

The data supporting the findings of this study are available on request from the corresponding author. The data is not publicly available due to ethical or privacy restrictions.

## Supplementary Materials

The following supporting information can be downloaded at: https://www.mdpi.com/article/10.3390/sports13100365/s1, Table S1: List of Articles Included in the Analysis. A complete list of articles that comprise the sample analyzed in this study is presented, including the sequential number, authors, title, journal, and year of publication. This dataset served as the basis for the subsequent scientometric analyses. Table S2: Frequency and Distribution of Keywords Extracted from Article Metadata. The terms identified through co-occurrence analysis of keywords from the included articles are presented, along with their frequency of occurrence.

## Abbreviations

The following abbreviations are used in this manuscript:

FIFA	International Federation of Association Football
WoS	Web of Science
n.d.	No Date
USP	University of São Paulo
UK	The United Kingdom
